# Minimally invasive versus open transforaminal lumbar interbody fusion

**DOI:** 10.4103/2152-7806.63905

**Published:** 2010-05-31

**Authors:** Alan T. Villavicencio, Sigita Burneikiene, Cassandra M. Roeca, E. Lee Nelson, Alexander Mason

**Affiliations:** 1 Boulder Neurosurgical Associates, Boulder, CO; USA; 2 Justin Parker Neurological Institute, Boulder, CO, USA; 3 University of Colorado School of Medicine, Aurora, CO, USA

**Keywords:** Clinical outcomes, Complications, Degenerative lumbar spine, Lumbar fusion, Minimally invasive approach, Open approach, Transforaminal lumbar interbody fusion

## Abstract

**Background:**

Available clinical data are insufficient for comparing minimally invasive (MI) and open approaches for transforaminal lumbar interbody fusion (TLIF). To date, a paucity of literature exists directly comparing minimally invasive (MI) and open approaches for transforaminal lumbar interbody fusion (TLIF). The purpose of this study was to directly compare safety and effectiveness for these two surgical approaches.

**Materials and Methods:**

Open or minimally invasive TLIF was performed in 63 and 76 patients, respectively. All consecutive minimally invasive TLIF cases were matched with a comparable cohort of open TLIF cases using three variables: diagnosis, number of spinal levels, and history of previous lumbar surgery. Patients were treated for painful degenerative disc disease with or without disc herniation, spondylolisthesis, and/or stenosis at one or two spinal levels. Clinical outcome (self-report measures, e.g., visual analog scale (VAS), patient satisfaction, and MacNab's criteria), operative data (operative time, estimated blood loss), length of hospitalization, and complications were assessed. Average follow-up for patients was 37.5 months.

**Results:**

The mean change in VAS scores postoperatively was greater (5.2 vs. 4.1) in theopen TLIF patient group (*P* = 0.3). MacNab's criteria score was excellent/good in 67% and 70% (*P* = 0.8) of patients in open and minimally invasive TLIF groups, respectively. The overall patient satisfaction was 72.1% and 64.5% (*P* = 0.4) in open and minimally invasive TLIF groups, respectively. The total mean operative time was 214.9 min for open and 222.5 min for minimally invasive TLIF procedures (*P* = 0.5). The mean estimated blood loss for minimally invasive TLIF (163.0 ml) was significantly lower (*P* < 0.0001) than the open approach (366.8 ml). The mean duration of hospitalization in the minimally invasive TLIF (3 days) was significantly shorter (*P* = 0.02) than the open group (4.2 days). The total rate of neurological deficit was 10.5% in the minimally invasive TLIF group compared to 1.6% in the open group (*P* = 0.02).

**Conclusions:**

Minimally invasive TLIF technique may provide equivalent long-term clinical outcomes compared to open TLIF approach in select population of patients. The potential benefit of minimized tissue disruption, reduced blood loss, and length of hospitalization must be weighted against the increased rate of neural injury-related complications associated with a learning curve.

## INTRODUCTION

Various lumbar interbody fusion approaches are utilized to treat degenerative disc disease and spinal instability. The transforaminal lumbar interbody fusion (TLIF) technique is a modification of posterior lumbar interbody fusion (PLIF) that allows a more lateralized, one-sided, and direct access to the intervertebral foraminal area without violation of the anatomical integrity of the spinal neural elements. Performed correctly, it requires less retraction of the nerve root and the thecal sac, offers the benefit of circumferential fusion and maintenance or regaining of lumbar lordosis.[16,18,22,27,35]Since it was first proposed in the 1980s, TLIF has been shown to be a safe and effective surgical procedure. [2,8,14,22,25,30,34,42]

Advances in image-guided surgery have expanded the ability to treat spinal disorders surgically in a less invasive fashion. Image-guided techniques and surgical navigation systems provide intraoperative visualization of three-dimensional relationships of multifaceted spinal structures, thus assisting in more precise and accurate surgery. [41] TLIF surgical intervention can utilize either an open or minimally invasive (MI) approach. The latter has been rapidly gaining popularity because of its minimized tissue trauma and its potential for less blood loss. [19,20,36,37] These prospective benefits of minimally invasive TLIF have been shown to result in decreased narcotic use, length of hospitalization, and quicker recovery. [1,6,9,15,20,36,37] 

Foley and Lefkowitz examined several commonly performed spinal surgical procedures and concluded that although they are “minimally invasive, they are not minimally effective.” [10]The rationale of this study was to directly compare effectiveness and safety for these two surgical approaches and to verify that by minimizing iatrogenic tissue injuries the same result can be accomplished without compromising the purported benefits of the surgery.

## MATERIALS AND METHODS

A total of 139 TLIF surgical cases consecutively performed between September 2002 and December 2004 were retrospectively analyzed using a retrospective case–control study design. Seventy-six minimally invasive TLIF cases were compared with a cohort of 63 consecutively performed open TLIF cases using three variables: diagnosis, number of spinal levels, and history of previous lumbar surgery. Patients were treated for painful degenerative disc disease with or without disc herniation, spondylolisthesis, and/or stenosis at one or two spinal levels. Prior to the surgery, extensive clinical and neurological evaluations were completed to assess possible motor, sensory, or reflex deficits. Radiological examination including lumbar spine magnetic resonance imaging (MRI) and/or computed tomography (CT), and plain radiographs were performed to confirm clinical diagnosis. One or more of the following had to be present in the radiographic findings in order to appropriate the diagnosis of degenerative disc disease: disc dehydration, decreased disc height, endplate destruction, Modic changes, and/or high-intensity zone lesions. Clinically relevant spinal levels were determined based on history, physical examination, and diagnostic studies. Provocative discography was infrequently utilized to identify a specific intervertebral disc space as a pain generator. MRI also assisted in determining whether or not there was neural compression owing to disc herniation and/or central stenosis. Lumbar instability was based on evidence of dynamic anterior-posterior translation of 4 mm or more and/or angulation greater than or equal to 10° on flexion-extension films. CT myelography was utilized to evaluate for neural compression in a minority of cases that were indeterminant on MRI. All patients in this study underwent conservative therapy for a minimum period of 6 months prior to the surgery unless their symptoms were progressive or existed in conjunction with major spinal instability confirmed in imaging studies. Conservative management included at least one of the following: anti-inflammatory medications, steroids, physical therapy, epidural steroid injections, and chiropractic care.

Patients with at least 24 months of follow-up were included in this study; the mean follow-up was 37.5 (range 26–52) months. Patient demographic data are presented in Table 1. Sixty-three patients (45%) were operated using an open approach, and another 76 patients (55%) underwent surgery using MI approach. Twenty-five out of 63 (39.7%) of the open and —21 out of 76 (28%) of the minimally invasive TLIF group patients had undergone previous lumbar surgeries that included discectomy, decompression surgeries, and fusion at the adjacent levels. Demographic characteristics were similar between the groups with respect to age, gender, the number of previous surgeries, and two-level procedures performed (*P* = 1.0, Chi-square test).

**Table 1:** Patient demographics

	Open	Minimally invasive
Patients	63	76
Average age	58.9 (30–86)	50.5 (19–91)
Gender (% male)	38	45
Previous surgeries	25 (40)	21 (28)
One-level procedures	47 (75)	57 (75)
Two-level procedures	16 (25)	19 (25)

Values are presented numbers, with percentages in parenthesis; age presented as number with range in parenthesis

### Surgical procedure

The surgical procedures for both open and MI approaches have been previously described. [42] Minimally invasive TLIF was executed using either the CD Horizon^®^ Sextant system (Medtronic Sofamor Danek, Memphis, TN) or PathFinder minimally invasive system (Spinal Concepts, Austin, TX). Intraoperative image and data transfer were obtained by utilization of the Stealth frame neuronavigational platform (Stealth NeuroStation, Sofamor Danek, MKM Zeiss) and Iso-C three-dimensional fluoroscopy (Siremobil Iso-C ^3D^, Siemens Medical Solutions, Erlangen, Germany) for the MI cases. Iso-C fluoroscopy functions concurrently as a regular fluoroscopy unit, while also permitting for three-dimensional reconstruction of images into axial, sagittal, and coronal planes. All pedicle screws were inserted using Iso-C fluoroscopy image guidance, and an additional intraoperative “spin” was performed to verify good screw placement for the minimally invasive TLIF group patients. Open TLIF surgeries were performed under conventional biplanar fluoroscopic guidance.

All patients underwent placement of interbody structural allografts (Lanx, Broomfield, CO; Medtronic Sofamor Danek, Memphis, TN; Spinal Concepts, Austin, TX) and locally harvested autograft from the hemilaminectomy/facetectomy defect. In some cases, cancellous bone substitute, ChronOS (Synthes) was utilized. Forty-three out of 63 (68.3%) patients in the open TLIF approach group and —61 out of 76 (80%) in the minimally invasive TLIF approach group received bone morphogenetic protein (rhBMP-2) (Medtronic Sofamor Danek, Memphis, TN) to induce fusion. We have previously reported that the number of treated spinal levels or approach used has not affected the efficacy of rhBMP-2-induced fusion. [42] Although 80% of patients in the MI approach group received rhBMP-2 compared to 68.3% of patients in the open group, this difference was not statistically significant (*P* = 0.12, Fisher's exact test). In the early cases, the decision to use rhBMP-2 was based on the patients' clinical history, i.e., smoking. In the latter cases, all patients were treated with rhBMP-2.

### Outcome measures

#### Effectiveness

Operative data, clinical outcome, and patient satisfaction were compared for open and minimally invasive TLIF procedures in order to evaluate effectiveness. Operative data such as duration of the procedure (OR time), estimated surgical blood loss (EBL), and length of stay (LOS) were assessed. Discharge criteria were based on patient clinical status, ambulation, and effective postoperative pain control.

Clinical outcome was evaluated using pre- and postoperative VAS scores to assess pain. MacNab's criteria were used to characterize patients' identifiable comprehensive outcome after TLIF surgery. [23] According to MacNab's criteria, the results were described as excellent (completely free from all pain), good (minor intermittent discomfort not interfering with normal activities), fair (improvement in symptoms, but persistent backache or sciatica interfering with capacity to engage in full normal activities), and poor (no change in symptoms). Excellent/good and fair/poor perceptions were combined for statistical analysis. Patient Satisfaction with Results Survey (PhDx Systems, Albuquerque, NM) was also conducted postoperatively. The detail of the questionnaire is presented in Table 2. Scores for each question were evaluated separately, and results were compared between the two approaches. Postoperative questionnaires were administered by an independent interviewer (CMR) who was not associated with patient care.

**Table 2:** Patient's satisfaction with results survey

	Open (%)	Minimally invasive (%)
I can do the things I thought I would be able to do after surgery	57.6	60.4
I was helped as much as I thought I would be by my surgery	68.0	59.9
My pain was reduced as much as I expected it to be after the surgery	67.3	60.4
The benefits of my care outweighed the setbacks it caused me	78.0	68.4
Overall I am happy with the care I am receiving for my lower back and/or legs	83.3	73.1
All things considered, I would have the surgery again for the same condition	78.1	64.6
Total (overall satisfaction)	72.1	64.5

Answers were scored on a scale from 0 to 100 : 100 = Definitely true; 75 = Mostly true; 50 = Don't know; 25 = Mostly false; 0 = Definitely false

Fusion was defined as an evidence of trabecular bone bridging on the CT scans and less than a 5° difference in angular motion between flexion and extension, and/or no radiolucency lines greater than 2 mm in thickness covering more than 50% of the superior or inferior surface of the grafts on the plain radiographs.

#### Safety

Clinical, neurological evaluation and radiological studies, including plain radiographs, CT and MRI scans if necessary, were used to assess complications. Complications were strictly monitored and categorized as either major or minor. The major complications group included pedicle screw or allograft malposition that required reoperation, new or increased neurologic deficit that lasted more than 3 months and notwithstanding substantial conservative treatment, infection, or other complications that required a patient's readmission to the hospital. Switching from a MI to open procedure was also included in the major complications group. The minor complications group included allograft or pedicle screw malposition that did not require reoperation, transient (< 3 months) neurologic deficit that was effectively treated conservatively including physical therapy and/or steroid injections. Cerebrospinal fluid (CSF) leak, hematoma, and anemia that did not require reoperation or readmission to the hospital were also included in the minor complications group.

### Statistical analysis

For descriptive purposes, quantitative data were presented as means (range) and qualitative data were expressed in percentages. *P* < 0.05 was considered significant. Analysis of categorical variables between the two procedures was done using Pearson's Chi-square test or Fisher's exact test. Continuous variables were analyzed using Students *t*-test.

## RESULTS

### Effectiveness

**Figure 1:**
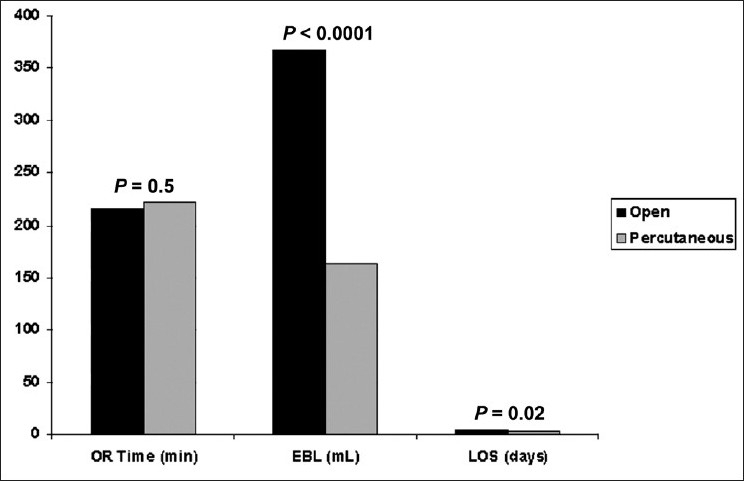
Operative data. OR time, operative time; EBL, estimated blood loss; LOS, length of stay

Operative data for open and minimally invasive TLIF surgical procedures are presented in Figure 1. The total operative time was comparable between the open and minimally invasive TLIF patient groups: 214.9 ± 60 and 222.5 ± 67.5 min, respectively. There was no statistically significant difference found in the mean operative times (*P* = 0.5, Student's *t*-test). Estimated blood loss for MI (163.0 ± 131.2 ml) was significantly lower (*P* < 0.0001, Student's *t*-test) than the open TLIF approach (366.8 ± 298.2 ml). The mean duration of hospitalization in the MI group (3.0 ± 2.3 days) was also significantly shorter (*P* = 0.02, Students *t*-test) than the open TLIF group (4.2 ± 3.5 days).

The total mean preoperative VAS scores were 8.0 and 7.4 (*P* = 0.3, Student's t-test) in the open and minimally invasive TLIF patient groups, respectively. Postoperative VAS scores were 3.2 and 3.4 (*P* = 0.8, Student's *t*-test) in the open and minimally invasive TLIF patient groups, respectively. The mean change in VAS scores postoperatively was greater in the open TLIF patient group (5.2 vs. 4.1), although this difference was not statistically significant (*P* = 0.3, Student's *t*-test).

Clinical outcome, defined by patients' perceived overall treatment effect (MacNab's criteria), was excellent/good in 67% and 70% of patients in open and minimally invasive TLIF groups, respectively. There was no statistical difference between the clinical outcome for these two procedures (*P* = 0.76, Fisher's exact test). The overall patient satisfaction was 72.1% and 64.5% in open and minimally invasive TLIF groups, respectively (*P* = 0.4, Fisher's exact test). Results for the individual questions of this survey are presented in Table 2.

Fusion was achieved in all patients based on the radiographic evidence described in “Materials and Methods” section. There were no instances of ectopic bone formation in subjects with rhBMP-2-induced fusion.

### Safety

The total complication rate was 31.6% in the minimally invasive TLIF group and 31.7% in the open TLIF group. Complications are reported as major [Table 3] or minor [Table 4] according to the criteria described in the “Materials and Methods” section. Patients in the open TLIF group had 22.2% minor and 9.5% major complications compared to 13.2% minor and 18.4% major complications in the minimally invasive TLIF group. Although it was not statistically significant (*P* = 0.15, Fisher's exact test), patients in the minimally invasive TLIF approach group had nearly double the rate of major complications.

**Table 3:** Major complications

	Open	Minimally invasive
Allograft malposition w/Re-op	2 (3.2)	3 (3.9)
Pedicle screw malposition w/Re-op	2 (3.2)	4 (5.3)
Infection	1 (1.6)	1 (1.3)
Neurological deficit (>3 mos)	1 (1.6)	5 (6.6)
Switch from percutaneous to open	N/A	1 (1.3)
Total	6 (9.5)	14 (18.4)

Values are given as numbers with percentages in parenthesis. w/Re-op, with reoperation; mos, months.

**Table 4:** Minor complications

	Open	Minimally invasive
Pedicle screw malposition	2 (3.2)	3 (3.9)
CSF leak	7 (11.1)	1 (1.3)
Neurological deficit (<3 mos)	None	3 (3.9)
Hematoma	2 (3.2)	2 (2.6)
Anemia	3 (4.8)	1 (1.3)
Total	14 (22.2)	10 (13.2)

Values are given as numbers with percentages in parenthesis. CSF, cerebrospinal fluid; mos, months.

Two patients (3.2%) in the open and three patients (3.9%) in the MI group were taken back to the operating room for repositioning or removal of malpositioned allografts. This included that one patient at 3 months post-open TLIF surgery was found to have an allograft that had migrated posteriorly at the L4-5 segment, causing lower-extremity radicular symptoms, was taken back to the operating room for removal of the allograft. Another patient in the open group required reoperation 5 months after TLIF surgery for removal of an allograft that protruded on the right side of the spinal canal causing right lateral recess stenosis at the L2 nerve root. Both these patients in the open TLIF are now reportedly asymptomatic. In the minimally invasive TLIF group, one of the patients was re-admitted to the hospital 3 months after the surgery for allograft repositioning; this patient had a 7-mm allograft protrusion causing left-lateral recess and spinal canal encroachment. Two other patients in the MI group had removal of displaced allograft, one with 9-mm and another with 7-mm right displacement that was causing foraminal stenosis with symptomatic nerve root compression. These patients in the minimally invasive TLIF group are now reportedly asymptomatic.

Two patients (3.2%) in the open and four patients (5.3%) in the minimally invasive TLIF groups required reoperation for pedicle screw repositioning or removal. Of these, two patients in the open group and two in the minimally invasive TLIF group had screws repositioned for lateral perforations of more than 4 mm. The remaining two patients in the MI group had a medial pedicle screw perforation, one 6-mm and one 5-mm, at L5 and underwent reoperation for screw removal. A postoperative “confirmation” spin with Iso-C fluoroscopy was not performed on these patients. For two patients in the open group and three patients in the MI group, asymptomatic lateral pedicle screw perforations of less than 4 mm were elected to be left in place. These patients have not experienced any symptoms due to the screw misplacement.

Any new or increased postoperative neurological deficit was included in complication analysis. The total rate of neurological deficit (including major and minor) was 10.5% in the minimally invasive TLIF group, which was significantly greater as compared to 1.6% in the open TLIF group (*P* = 0.02, Fisher's exact test). There were three patients (3.9%) in the MI group with transient neurological deficit that lasted <3 months. These patients were effectively treated conservatively with physical therapy and/or steroid injections. One patient in the open and five in minimally invasive TLIF group experienced neurological deficit that lasted longer than 3 months notwithstanding substantial conservative treatment.

Three patients in the open group and one patient in the MI group had anemia. Of these patients, two in the open group and the one patient in the MI group received blood transfusions. Seven patients (11.1%) in the open TLIF group and one (1.3%) in the minimally invasive TLIF group had CSF leaks (*P* = 0.005, Fisher's exact test). CSF leaks were mended intraoperatively with 5-0 Prolene sutures and/or Gelfoam and two-component fibrin sealant Tisseal (Baxter AG, Vienna, Austria). Two patients in each the MI and open groups, 2.6% and 3.2%, respectively, had hematomas that resolved spontaneously. Infections were effectively treated with antibiotics in both patient groups.

## DISCUSSION

Varieties of lumbar spine fusion techniques have been modified with endoscopic or MI alternatives in an attempt to decrease the invasiveness of traditional approaches. [3,7,9,12,13,21,29,31,43] Khoo *et al*. demonstrated that a PLIF procedure could be safely and effectively performed using MI techniques. [21] Gepstein *et al*. compared the results of minimally invasive PLIFs using B-twin expandable spinal spacers with a technique in which the spacers were introduced via an open approach. [12] They found that although they had comparable clinical outcomes, the circumstances of the less-tissue traumatic MI approach were favored. Escobar *et al*. retrospectively analyzed the results of four different clinical approaches for anterior lumbar interbody fusion (ALIF). [7] It was concluded that the transperitoneal insufflation and retroperitoneal gasless video-assisted approaches should be abandoned due in part to an increased incidence of retrograde ejaculation and increased operating time compared to the traditional flank and minilaparotomy open techniques. Thus, although novel and less-invasive techniques appear theoretically attractive and feasible, thorough analysis and direct comparison with the corresponding time-tested open approach must be taken to ensure safety and undiminished quality of the outcome of the procedure.

Previous publications evaluated safety and effectiveness of different minimally invasive TLIF procedures. Isaacs *et al*. compared the safety of a microendoscopic TLIF (METLIF) and open PLIF. [19] Patients that underwent surgery with the METLIF approach had significantly lower EBL, LOS, and narcotic usage, with no short-term procedure-related complications. Deutsch *et al*. evaluated clinical outcomes of 34 patients who underwent MI one-level TLIF. [6] Seventeen of these patients (85%) had a good clinical outcome, indicated by a significant (more than 20-point reduction) improvement of Oswestry Disability Index (ODI) score. A minimally invasive TLIF utilizing ipsilateral pedicle screw and contralateral facet screw fixation was introduced by Jang and Lee. [20] A recently published study compared the results of a MI technique with a historical cohort of patients that had undergone surgery using mini-open TLIF approach. [36] There were no statistically significant differences between the two groups with respect to the overall clinical outcome and OR time (which was even shorter in the MI performed surgeries); however, blood loss was significantly reduced in the MI group. The main benefit that was highlighted in this paper was that the MI technique significantly reduced postoperative access site pain, which lead to decreased use of analgesics.

As with any surgical procedure, MI surgery has potential limitations and drawbacks. Schwender *et al*. pointed out some disadvantages of the minimally invasive TLIF approach: anatomic disorientation due to unexposed landmarks and smaller working area requiring longer bayoneted instruments. [37] Ozgur *et al*. noted the difficulty that is accompanied by MI exposure in the incidence of repairing a CSF leak or capacious bleeding. [28] The authors of this study believe that there is a substantial learning curve associated with the increased rate of approach related neurological complications.

Thus, although there is increasing literature reporting new minimally invasive TLIF techniques, comprehensive and careful assessment is required that takes into account not only immediate perioperative advantages, but also safety and long-term clinical outcomes. [1,6,15,19,36,37,39] 

### Effectiveness

Two clinical parameters, EBL and OR time, could aid in describing the effectiveness as well as give some insight into the technical complexity of the minimally invasive TLIF procedure. Reduced blood loss can be expected in the MI approach, as muscle stripping required in the open approach becomes negligible with a minimal incision and employment of the spinal instrumentation used to access the bony anatomy. Previously published data report estimated blood loss averages for MI and open procedures ranging from 50 to 310 ml, and 378 to 1070 ml, respectively. [1,4,6,19,20,36,37,39,42,44] This study reported an estimated blood loss of 163.0 (range, 25–750) ml for the MI approach, which was significantly lower (*P* < 0.0001, Students *t*-test) than the 366.8 (range 25-2500) ml for the open approach. Although it took slightly longer to perform the minimally invasive TLIF procedure, the authors of this study found no statistically significant difference between the operative times in either group. Operative time for MI and open TLIF approaches reported in this study was 222.5 (range 114–370) min and 214.9 (108–517) min, respectively. It has to be taken into account that Iso-C fluoroscopy was used in all MI performed TLIF cases, which adds additional time to the total OR time calculated. Previously published data report the mean operative times for MI and open procedures, ranging from 150 to 300 min and 144.4 to 192 min, respectively. [1,18,19,20,37,44] Scheufler *et al*. reported shorter OR times for one-level percutaneous compared to mini-open TLIF procedure: 104 ± 17 vs. 132 ± 18 min, respectively. [36] 

Approach-related reduction of tissue morbidity theoretically should shorten both length of hospitalization and recovery time in general. A significantly shorter length of hospitalization for the minimally invasive TLIF group compared to the open group patients was observed in this study: 3.0 (1–16) days and 4.2 (1–24) days, respectively. This is consistent with the literature, which reports average hospitalization length ranges of 4.1–5.1 and 1.6–3.4 days for open and minimally invasive TLIF approaches, respectively. [1,4,6,19,26,37,39,42,44] These results suggest practical advantages of minimized tissue injury in the minimally invasive TLIF approach with regards to recovery. Indeed, the increased duration of muscle compression, independent of the intramuscular pressure delivered by the type of retractor utilized, has been linked to greater ischemic damage and a longer period of recovery. [5,11,38] Clinical assessment of postoperative pain, function, and health using the VAS, ODI, and Short Form 36 (SF-36) scores has shown score improvement to be directly linked to a shorter duration of intramuscular pressure. [5] 

Although the cost benefits of the minimally invasive TLIF approach have been questioned because of the specialized and expensive instruments that this type of lumbar fusion demands, it could be at least partially offset by the shorter hospitalization and less narcotic use that accompanies a quicker recovery. [7,9,19] 

The primary clinical outcomes in this particular study were measured as the pre- and postoperative VAS scores. Average VAS scores prior to and after surgery were similar between both groups. The mean change in VAS scores postoperatively was greater in the open TLIF patient group (5.2 vs. 4.1) at the mean follow-up time of 37.5 months, although this difference was not statistically significant. These results demonstrate that both surgical approaches provide similar pain relief. Other clinical outcomes used in this study included patients' perceived global outcome (MacNab's criteria), and patient satisfaction (Patient Satisfaction with Results Survey). This study found no significant difference between the open and minimally invasive TLIF approaches with regards to MacNab's criteria or patient satisfaction.

With no significant difference in clinical outcomes or operative time, both open and minimally invasive TLIF procedures appear to be equally effective. Several benefits accompany the minimally invasive TLIF approach including reduced length of hospitalization and decreased blood loss.

### Safety

Some of the most frequently encountered complications that have previously been reported for the open TLIF procedure include allograft and screw malpositioning, deep vein thrombosis, CSF leak, ileus, blood vessel damage, infection, pseudoarthrosis, neurological deficit, and pulmonary embolism. [8,24,30,34,42] This study presented a total complication rate of 31.7% for open TLIF procedures, which is slightly higher than previously reported open TLIF complication rates, ranging from 16.1% to 27.3%. [8,22,30,34,35,44] 

Complications in the MI approach are similar, and previously reported data have included CSF leak, nerve root injury, malpositioned hardware, infection, pseudoarthrosis, hematoma, and converting from MI to open procedure. [6,26,37,39,40] For minimally invasive TLIF procedures in this study, the total complication rate of 31.6% is also higher than that reported in the literature, which range from 0 to 19.1%. [1,6,9,19,28,36,37,39] 

There was no statistically significant difference between the total rate of complications reported in this study for the MI and open TLIF groups. However, there is a clinically significant difference with a higher rate of major complications in the MI approach. The open TLIF patient group had a significantly higher incidence of CSF leaks compared to the MI group (1.1% and 1.3%, respectively), which may be related to the increased number of redo operations in the open TLIF group since five out of seven (71%) leaks in the open group occurred in the patients who underwent previous surgeries.

### Learning curve

In general, there is a learning curve associated with the development of proficient skills to enable a surgeon to perform the procedure safely and effectively. We believe that the most important drawback encountered was the incidence of neural injury complications initially. Total neurological deficit in the minimally invasive TLIF group was 10.5% and significantly greater than the 1.6% rate of occurrence in the open group. This demonstrates over a major increase in neural complications in the MI group. However, through sufficient experience with the MI technique, the rate of neural injury complications is diminished as six out of eight of these injuries occurred within the first 15 MI procedures performed. Thus, the increased occurrence in the minimally invasive TLIF group can be attributed to the substantial learning curve accompanied by the minimally invasive TLIF approach.

The considerable learning curve associated with minimally invasive TLIF also affects operative time and blood loss. Regan *et al*. directly compared laparoscopic vs. open ALIF approaches and concluded that the laparoscopic ALIF approach is conjoined by a learning curve of 5-10 cases. [32] They analyzed the experiences of eight different surgeons and reported a significant reduction in a mean operative time of nearly 1 h between the first and last performed cases. Our results demonstrate the same tendency that affects operative time and estimated blood loss in the minimally invasive TLIF procedure. The mean operative time for the first 26 MI cases performed was 238.5 min. Within the middle 25 and the last 25 cases performed, patients' average operative time dropped to 231.5 min (*P* = 0.7, Chi-square test) and 193.2 min (*P* = 0.015, Chi-square test), respectively. The estimated blood loss diminished over time as well, although only in the last 25 cases performed. The first 26 MI cases had an estimated blood loss of 175.0 ml. The middle 25 cases did not change from this value, and the last 25 cases decreased in blood loss to 107.61 ml (*P* = 0.037, Chi-square test). These results are consistent with those previously reported for the minimally invasive TLIF approach, and demonstrate that sufficient experience with this technique is required for maximized results. [21,29] 

### Limitations

The main limitation of this study is that the design was essentially a retrospective analysis, therefore a proper design (e.g., randomization) and standardized clinical outcome assessment tools were not utilized. We must agree that prospective trial produces superior clinical data, but restrictions placed by rigorous inclusion criteria and indications may not represent a typical clinical practice. In addition, we recognize that MacNab criteria as a clinical outcome assessment tool has not been validated, but regardless are widely used for retrospective and even prospective clinical studies. MacNab's criteria was recently utilized in a prospective study of patients undergoing lumbar disc herniation surgery that demonstrated a high agreement between patient-reported outcome and objective outcome 2 years after surgery. [33] Furthermore, utilizing patients' satisfaction with treatment questionnaires has been a commonly used instrument for outcome evaluation, and some have suggested this to be the most essential outcome measurement. [17] Despite the nature of this research, standardized pre- and postoperative VAS assessments were available, as it is customary for surgeons in this practice to utilize this pain scale with patients pre- and postoperatively.

The study was not randomized, and we have to acknowledge that this is the most important limitation of this analysis. However, as much as we hoped to demonstrate the superiority of this novel MI approach—we could not do that in terms of safety, clinical outcomes, and patient satisfaction with this procedure. There were no statistically significant differences between the groups with respect to diagnosis, number of spinal levels, and history of previous lumbar surgery, but even if these differences that were not statistically significant have been taken into account, one would expect that patients in the open TLIF group had less favorable clinical outcomes. On the contrary, patients in the open TLIF group had greater VAS score change and overall satisfaction postoperatively than the minimally invasive TLIF group. Therefore, on the basis of the results of this study, it is safe to say that minimally invasive TLIF technique is not superior compared to the open approach. The potential benefits of less blood loss and a faster recuperation appear to be offset by a higher rate of neurological complications.

## CONCLUSION

The minimally invasive TLIF technique may provide equivalent long-term clinical outcomes compared to open TLIF approach in select population of patients. The potential benefit of minimized tissue disruption, reduced blood loss, and length of hospitalization must be weighted against the increased rate of neural injury-related complications associated with a learning curve.
